# Strain on Scarce Intensive Care Beds Drives Reduced Patient Volumes, Patient Selection, and Worse Outcome: A National Cohort Study*

**DOI:** 10.1097/CCM.0000000000006156

**Published:** 2023-12-14

**Authors:** Sylvia Brinkman, Nicolette F. de Keizer, Dylan W. de Lange, Dave A. Dongelmans, Fabian Termorshuizen, Bas C.T. van Bussel

**Affiliations:** 1 Department of Medical Informatics, Amsterdam UMC Location University of Amsterdam, Amsterdam, The Netherlands.; 2 National Intensive Care Evaluation Foundation, Amsterdam, The Netherlands.; 3 Amsterdam Public Health, Quality of Care, Amsterdam, The Netherlands.; 4 University Medical Center, University of Utrecht, Intensive Care Medicine, Utrecht, The Netherlands.; 5 Amsterdam UMC Location University of Amsterdam, Intensive Care Medicine, Amsterdam, The Netherlands.; 6 Department of Intensive Care Medicine, Maastricht University Medical Centre +, Maastricht, The Netherlands.; 7 Cardiovascular Research Institute Maastricht (CARIM), Maastricht University, Maastricht, The Netherlands.; 8 Care and Public Health Research Institute (CAPHRI), Maastricht University, Maastricht, The Netherlands.

**Keywords:** critical care, intensive care, mortality, non-COVID-19, overload, pandemic, strain, surge

## Abstract

**OBJECTIVES:**

Strain on ICUs during the COVID-19 pandemic required stringent triage at the ICU to distribute resources appropriately. This could have resulted in reduced patient volumes, patient selection, and worse outcome of non-COVID-19 patients, especially during the pandemic peaks when the strain on ICUs was extreme. We analyzed this potential impact on the non-COVID-19 patients.

**DESIGN:**

A national cohort study.

**SETTING:**

Data of 71 Dutch ICUs

**PARTICIPANTS:**

A total of 120,393 patients in the pandemic non-COVID-19 cohort (from March 1, 2020 to February 28, 2022) and 164,737 patients in the prepandemic cohort (from January 1, 2018 to December 31, 2019).

**INTERVENTIONS:**

None.

**MEASUREMENTS AND MAIN RESULTS:**

Volume, patient characteristics, and mortality were compared between the pandemic non-COVID-19 cohort and the prepandemic cohort, focusing on the pandemic period and its peaks, with attention to strata of specific admission types, diagnoses, and severity. The number of admitted non-COVID-19 patients during the pandemic period and its peaks were, respectively, 26.9% and 34.2% lower compared with the prepandemic cohort. The pandemic non-COVID-19 cohort consisted of fewer medical patients (48.1% vs. 50.7%), fewer patients with comorbidities (36.5% vs. 40.6%), and more patients on mechanical ventilation (45.3% vs. 42.4%) and vasoactive medication (44.7% vs. 38.4%) compared with the prepandemic cohort. Case-mix adjusted mortality during the pandemic period and its peaks was higher compared with the prepandemic period, odds ratios were, respectively, 1.08 (95% CI, 1.05–1.11) and 1.10 (95% CI, 1.07–1.13).

**CONCLUSIONS:**

In non-COVID-19 patients the strain on healthcare has driven lower patient volume, selection of fewer comorbid patients who required more intensive support, and a modest increase in the case-mix adjusted mortality.

**KEY POINTS**

**Question:** Did strain on ICUs during the COVID-19 pandemic result inpatient selection and worse outcome of non-COVID-19 ICU patients?

**Findings:** National cohort study comparing characteristics and mortality of 120,393 non-COVID-19 patients admitted during the COVID-19 pandemic and 164,737 patients admitted to the ICU before the COVID-19 pandemic. The non-COVID-19 pandemic cohort patients had fewer comorbidities, required more intensive support, and had a modest increase in case-mix adjusted mortality.

**Meaning:** The COVID-19 pandemic had considerable impact on Dutch non-COVID-19 ICU care, suggesting that the highly efficient healthcare systems as in the Netherlands, do not have the capacity to cope with great surges in the number of ICU admissions.

To achieve efficient healthcare, in particular with respect to expensive ICUs, resource optimization is crucial. In the Netherlands the number of ICU beds had been limited to a ratio of 6.4 per 100,000 inhabitants, which is similar to the United Kingdom but lower than neighboring countries (1). However, this efficiency can strain capacity during emergencies like epidemics, as seen during the COVID-19 pandemic when Dutch ICU capacity increased from 1100 to 1700 beds in April 2020 (2). Hospitals adopted a crisis strategy that involved, amongst others, the reallocation of personnel and repurposing of surgical theaters and postoperative recovery areas into ICUs (3). The ICUs were forced to alter regular nursing and staff ratios and to bypass the Dutch Guidelines for Intensive Care stating that an ICU nurse in the Netherlands takes care for one or two patients per shift (4). Despite receiving assistance from non-ICU nurses for basic care and support from other nonintensivist physicians, both ICU nurses and intensivists remained responsible for the care and welfare of a substantially greater number of patients throughout their shifts, resulting in an increased workload per caregiver (5). This strain on ICUs might have impacted the care of non-COVID-19 ICU patients.

During the pandemic, the main focus was on accommodating COVID-19 patients, potentially impacting non-COVID-19 care. These impacts include: reduced elective surgeries (3, 6, 7), lower emergency department utilization (8–10), and decreased medical consultations (11). However, uncertainties regarding types of admission, diseases, and severity strata during the pandemic period and its peaks for all ICU patients within the Dutch healthcare system remain. During the peak weeks of the pandemic the strain of ICUs was most extreme. Therefore, any effects of strain upon non-COVID-19 patients would probably be more easily detected during these peak weeks. Furthermore, impact might be specific for certain diagnoses or disease severity strata (12, 13).

To assess healthcare strain, we studied all Dutch non-COVID-19 ICU patients during the pandemic and compared them to a prepandemic cohort. We hypothesized that stricter patient selection led to fewer non-COVID-19 admissions and poorer outcomes during the pandemic. We also explored differences among medical and surgical patients, diagnoses, and severity. Additionally, we analyzed whether ICU occupancy was associated with increased in-hospital mortality for non-COVID-19 patients.

## MATERIALS AND METHODS

In this national cohort study the National Intensive Care Evaluation Database (NICE-DB), which is operational since 1996, was used. The NICE-DB contains, amongst others, demographic, physiologic, clinical, ICU and in-hospital mortality data of all consecutive patients admitted to all Dutch ICUs (14). First, all data were used to analyze the average occupancy ratio per week in the Dutch ICUs during the COVID-19 pandemic to illustrate the degree of strain on the ICUs. Furthermore, as the different variants of severe acute respiratory syndrome coronavirus 2 (SARS-CoV-2) have their unique properties, which influence infectivity and impact the health outcomes of patients, we also illustrated the most dominant variant present across the pandemic.

During the COVID-19 pandemic all COVID-19 patients admitted to the ICU were registered separately in the COVID-Database of NICE on a daily basis to monitor the course of the outbreak. Using this separate registration module, we identified several peak weeks of the pandemic in which there was an increased number of admissions due to COVID-19. The peak weeks were defined as calendar weeks with in total greater than 200 COVID-19 patients present at all Dutch ICUs. We observed four peaks as follows:

- First peak: from March 16, 2020 (week 12) to May 24, 2020 (week 21).
- Second peak: from October 5, 2020 (week 41) to June 20, 2021 (week 24).
- Third peak: from August 2, 2021 (week 31) to September 19, 2021 (week 37).
- Fourth peak: from October 25, 2021 (week 43) to February 6, 2022 (week 5).

Next, all patients who were considered to have COVID-19 based on a positive reverse transcriptase-quantitative polymerase chain reaction of their respiratory secretions for SARS-CoV-2 or a chest CT scan consistent with COVID-19 were excluded from the present study. In contrast, we included all patients admitted to Dutch ICUs who were classified as not having COVID-19 (i.e., non-COVID-19 patients) and used data from March 1, 2020 to February 28, 2022 (the 2 years COVID-19 pandemic period). In addition, as a historic control cohort we defined a prepandemic cohort including all patients admitted to Dutch ICUs. To exclude the possibility of COVID-19, we chose January 1, 2018 to December 31, 2019, similarly covering 2 full years, instead of March 1, 2018 to February 28 2020.

To investigate the effect on non-COVID-19 ICU admissions during the peak weeks, we compared the calendar weeks of all four peaks with the same calendar weeks (but 2 years earlier) in the prepandemic ICU cohort. As we use the same calendar weeks from the prepandemic period, the reference cohort covers the same seasons and therewith rules out potential differences in outcome due to seasonal influences.

We compared the volume, characteristics, and outcomes of the non-COVID-19 ICU patient cohort with the prepandemic ICU cohort, for the pandemic 2 years, and separately for the combined peak weeks. Additionally, we investigated the effect separately for medical, urgent surgery, and elective surgery patients, and for the following seven disease strata based on the Acute Physiologic Assessment and Chronic Health Evaluation (APACHE)-IV reason for ICU admission diagnosis (15): coronary artery bypass grafting (CABG) patients, out-of-hospital cardiac arrest patients, patients with a subarachnoid hemorrhage, oncology patients, trauma patients, intoxicated patients, and sepsis patients. Furthermore, we investigated three disease severity strata, using three separate APACHE-IV mortality risk groups (i.e., low < 20%, medium 20–70%, and high > 70% mortality risk).

The institutional research board (IRB) of the Amsterdam University Medical Centre reviewed the research proposal with the title “Impact of the COVID-19 pandemic on Dutch ICU non-COVID-19 patients; a national cohort study on volume, patient characteristics, and outcome” and waived the need for informed consent on November 10, 2022 (IRB protocol W22_394 number 22.468).

### Statistical Analyses

#### Degree of Strain on ICUs in Terms of Occupancy Ratio

Although strain on ICUs consists of several factors (e.g., increased stress level of employees, increased nursing patient ratio and nursing workload, shortages of medical equipment, and/or ICU beds), the occupancy ratio may reflect a combination of the most important components of strain. The occupancy ratio per day during the COVID-19 pandemic is estimated as the observed number of occupied ICU beds divided by the expected number of occupied ICU beds using the data of the years 2018 and 2019 (*O*_ob_/*E*_ob_) ratio. The observed number of occupied ICU beds refers to the actual number of ICU beds in a specific ICU that were occupied by (COVID-19 or non-COVID-19) patients on a specific day. The expected number was estimated for each ICU separately by taking the average number of occupied beds per day in 2018 and 2019 for that ICU (i.e., each ICU has his own reference value). This expected number of occupied ICU beds serves as a reference for assessing how the pandemic occupancy compares to prepandemic data. An *O*_ob_/*E*_ob_ ratio greater than 1 means that the number of occupied ICU beds is higher than expected during 2018 and 2019, whereas an *O*_ob_/*E*_ob_ ratio less than 1 means that there were fewer occupied ICU beds.

#### Differences in Non-COVID-19 Patient Volumes and Characteristics

With regard to non-COVID-19 patient volumes, the differences in the number of admitted non-COVID-19 cohort patients and prepandemic cohort patients were illustrated by calculating the observed number of newly admitted non-COVID-19 patients per calendar week during the pandemic period divided by the expected number of newly admitted patients expressed as the mean of the newly admitted patients in the corresponding calendar weeks in 2018 and 2019 (*O*/*E*) ratio. The *O*/*E* ratio of the total population was illustrated per week, and the *O*/*E* ratios for the defined diagnoses and severity strata were separately presented for the pandemic period and its peak weeks.

Both the pandemic non-COVID-19 patient cohort and the prepandemic patient cohort were investigated to compare the strain situation during the pandemic with the prepandemic intensive care situation in terms of important patient characteristics; age, gender, admission type (medical, urgent/elective surgery), chronic comorbidities, mechanical ventilation in the first 24 hours of ICU admission, vasoactive medication in the first 24 hours of ICU admission, the disease severity expressed as the APACHE-III score and APACHE-III Acute Physiology Score (APS) (16), and the length of ICU and hospital stay in days.

#### Differences in the In-Hospital Mortality

The in-hospital mortality among the non-COVID-19 patients was compared with that of the prepandemic patient cohort. We used mixed-effects logistic regression models with a random intercept per hospital, to account for the clustering of patients within hospitals, and calculated the odds ratio (ORs) and corresponding 95% CI.

First, the crude ORs of the in-hospital mortality were assessed in the total population and the defined subgroups. Second, to take important clinical differences between the two cohorts that may confound the association between the cohort and observed in-hospital mortality into account, the mixed-effects logistic regression models were adjusted for: age (categorized in 11 age groups, and a separate category for the patients with an unknown age), gender, body mass index (BMI) (categorized in the six internationally defined BMI groups (17), and a separate category for the patients with an unknown BMI), comorbidities present before hospitalization (i.e., immunological insufficiency, chronic renal failure, chronic respirator insufficiency or chronic obstructive pulmonary disease, chronic cardiovascular insufficiency, cirrhosis, malignancy, and diabetes mellitus), the APS of the APACHE-III prognostic model (APACHE-III APS) that quantifies the severity of physiologic disturbance in the first 24 hours of ICU admission (categorized in five groups based on quintiles of the APACHE-III APS) (15, 16), mechanical ventilation in the first 24 hours of ICU admission, and the use of vasoactive medication in the first 24 hours of ICU admission.

Third, the mixed-effects logistic regression models were further extended to incorporate the occupancy ratio at the ICU on the day when the patient was admitted to that ICU. The calculated occupancy ratio (*O*_ob_/*E*_ob_) was included as category with five groups based on the quintiles of the occupancy ratio. Additionally, the ORs and corresponding 95% CIs for the quintiles of occupancy ratio were extracted from this third model to assess the influence of the occupancy ratio on the in-hospital mortality of non-COVID-19 patients.

All analyses were performed using the R statistical environment (version 3.6.1; R Foundation for Statistical Computing, Vienna, Austria).

## RESULTS

### Degree of Strain on ICUs in Terms of Occupancy Ratio

**Figure 1.**
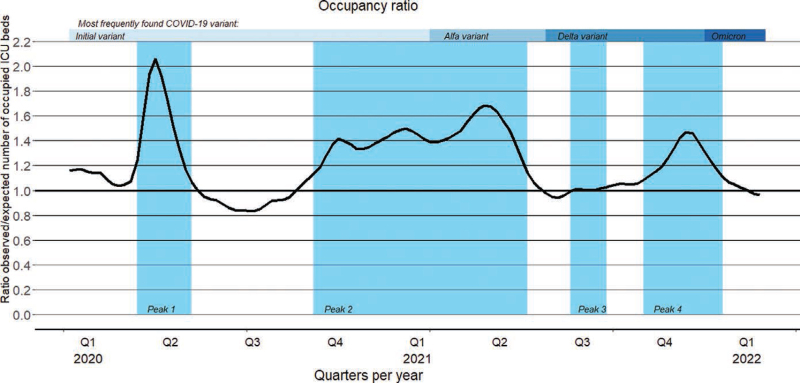
Occupancy ratio during the pandemic; expressed as the average of the observed number of occupied ICU beds divided by the expected average number of occupied beds in the years 2018 and 2019 per ICU (observed number of admissions/expected number of admissions ratio).

**Figure 1** illustrates the occupancy during the pandemic expressed as the *O*_ob_/*E*_ob_ ratio. It shows an overall higher occupancy during the COVID-19 pandemic compared to the prepandemic period. During the COVID-19 period there were on average 14.9% more occupied beds within the Dutch ICUs, whereas in peak weeks of the pandemic there were on average 34.9% more occupied beds within the ICUs. **Figure S1** (http://links.lww.com/CCM/H469) details the absolute number of occupied beds within ICUs during the study period. During the pandemic period, 58.1% of the occupied beds at the ICU were used by non-COVID-19 patients and during the peak weeks of the pandemic 47.9% of the occupied beds were used by non-COVID-19 patients. Figure 1 also illustrates the evolution of the predominant SARS-CoV-2 variant over time, showcasing the initial identification of the mutated variant in early 2021 (calendar week 7).

### Differences in Non-COVID-19 Patient Volumes and Characteristics

**Figure 2.**
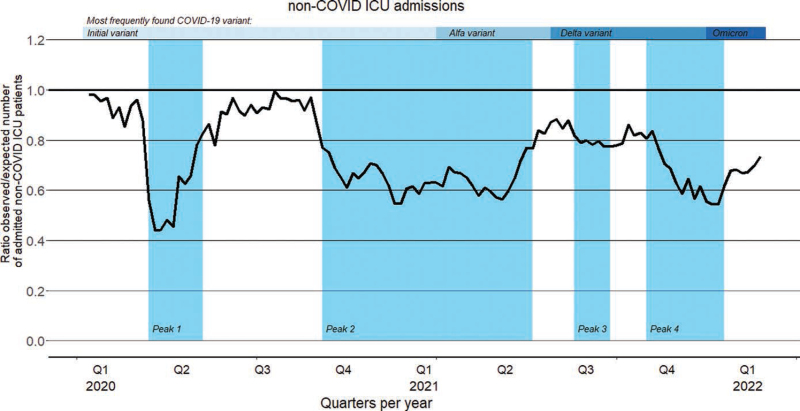
Differences in number of admitted non-COVID-19 patients expressed as observed number of admissions/expected number of admissions (*O*/*E*) ratio.

Of 285,130 patients admitted to the 71 Dutch ICUs within the 4-year study period, 120,393 (42.2%) were admitted as non-COVID-19 patients during the two pandemic years, of whom 72,173 patients (59,9%) were admitted during the peaks of the pandemic (**Fig. S2**, http://links.lww.com/CCM/H469). The number of admitted non-COVID-19 patients during the pandemic period was lower than the number of patients admitted during the prepandemic period (**Fig. 2**). During the peak weeks of the pandemic, that is, the blue shaded periods in the graph, the decline in the number of newly admitted non-COVID-19 patients was even greater. This decline was present in all analyzed disease and severity strata, that is, based at admission type, diseases, and APACHE-IV mortality risk (**Table 1**). The non-COVID-19 patients were less frequently medical patients (48.1% vs. 50.7%) and had fewer chronic comorbidities (36.5% vs. 40.6%) compared with prepandemic patients. Furthermore, the non-COVID-19 patients were more often mechanically ventilated (45.3% vs. 42.4%) and more often received vasoactive medication (44.7% vs. 38.4%) compared with prepandemic patients (**Table 2**). However, the overall disease severity and in-hospital mortality were similar. The in-hospital mortality in the prepandemic cohort was 12.0% and 12.2% during the weeks in 2018–2019 that corresponded to the pandemic period and its peaks, compared with, respectively, 12.3% and 12.9% in the pandemic cohort (Table 2). In **Tables S1–S13** (http://links.lww.com/CCM/H469), the demographics of the defined subgroups are presented.

**TABLE 1.** *O*/*E* Ratio Between the Non-COVID-19 and Prepandemic Patient Cohorts During the Pandemic Period and its Peaks, for all Patients and According to the Admission Types, Disease Strata, and Acute Physiology and Chronic Health Evaluation IV Mortality Risk Strata

Dutch ICU Patients	*O*/*E* Ratio During Pandemic Period^a^	*O*/*E* Ratio During Peaks of the Pandemic^a^
All non-COVID-19 patients	0.73	0.66
Admission type strata		
Medical	0.69	0.62
Urgent surgery	0.78	0.73
Elective surgery	0.74	0.67
Disease strata		
Coronary artery bypass graft	0.93	0.88
Out-of-hospital cardiac arrest	0.84	0.81
Subarachnoid hemorrhage	0.88	0.83
Oncology	0.70	0.65
Trauma	0.74	0.66
Intoxication	0.80	0.72
Sepsis	0.64	0.59
Acute Physiology Assessment and Chronic Health Evaluation IV mortality risk strata		
Low (< 20%)	0.76	0.69
Medium (20–70%)	0.71	0.65
High (≥ 70%)	0.75	0.71

*O*/*E* ratio = observed number of newly admitted patients divided by the expected number of newly admitted patients.

**TABLE 2.** Demographics of the Non-COVID-19 and Prepandemic Patient Cohorts, Admitted During the COVID-19 Pandemic Period and its Peaks

	Pandemic Period: Reference	Pandemic Period: Non-COVID-19	Peaks of Pandemic: Reference	Peaks of Pandemic: Non-COVID-19
Number of patients	164,737	120,393	109,741	72,173
Age, median (IQR)	66 (54–75)	66 (54–74)	66 (54–75)	66 (55–74)
Male (%)	60.0	61.6	60.0	62.0
Admission type (%)				
Medical admission	50.7	48.1	50.9	48.3
Urgent admission	11.5	12.3	11.5	12.8
Elective admission	32.2	32.5	32.0	32.5
Unknown	5.6	7.1	5.6	6.4
Comorbidities (%)				
Malignancy	6.5	5.6	6.4	5.6
Immunological	9.3	7.6	9.3	7.7
Respiratory insufficiency	15.7	11.5	15.8	11.3
Renal insufficiency	6.6	4.7	6.6	4.7
Cardiovasculair insufficiency	4.6	3.9	4.6	3.9
Liver cirrhosis	2.0	1.3	2.0	1.3
Diabetes mellitus	16.5	15.6	16.4	15.7
Patients with ≥ 1 comorbidities	40.6	36.5	40.8	36.6
Supportive treatment (%)				
Mechanically ventilated in first 24 hr of ICU admission	42.4	45.3	42.5	47.1
Vasoactive medication	38.4	44.7	38.6	46.6
Disease severity scores median (IQR)				
APACHE-III APS	36 (23–55)	37 (23–55)	36 (23–55)	37 (24–57)
APACHE-III score	49 (33–69)	49 (33–69)	49 (33–70)	50 (34–70)
APACHE-IV mortality risk strata (%)				
Low (< 20%)	74.0	75.1	73.6	74.4
Medium (20–70%)	20.5	19.3	20.8	19.8
High (≥ 70%)	5.6	5.5	5.6	5.8
Disease strata (%)				
Coronary artery bypass graft	9.3	11.8	9.2	12.3
Out-of-hospital cardiac arrest	3.4	3.9	3.4	4.2
Subarachnoid hemorrhage	0.6	0.7	0.6	0.8
Oncology	7.5	7.1	7.4	7.3
Trauma	4.4	4.5	4.2	4.2
Intoxication	3.9	4.2	3.8	4.1
Sepsis	6.1	5.3	6.0	5.4
Hospital mortality (%)	12.0	12.3	12.2	12.9
Length of ICU stay, median (IQR)	1 (0.7–2.6)	1 (0.7–2.2)	1 (0.7–2.7)	0.9 (0.7–2.1)
Length of hospital stay, median (IQR)	7.6 (4–14.8)	7 (4–14)	7.6 (4–15)	7.5 (4–14.4)

APACHE = Acute Physiology Assessment and Chronic Health Evaluation, APS = Acute Physiology Score, IQR = interquartile range.

### Differences in the In-Hospital Mortality

**Figure 3.**
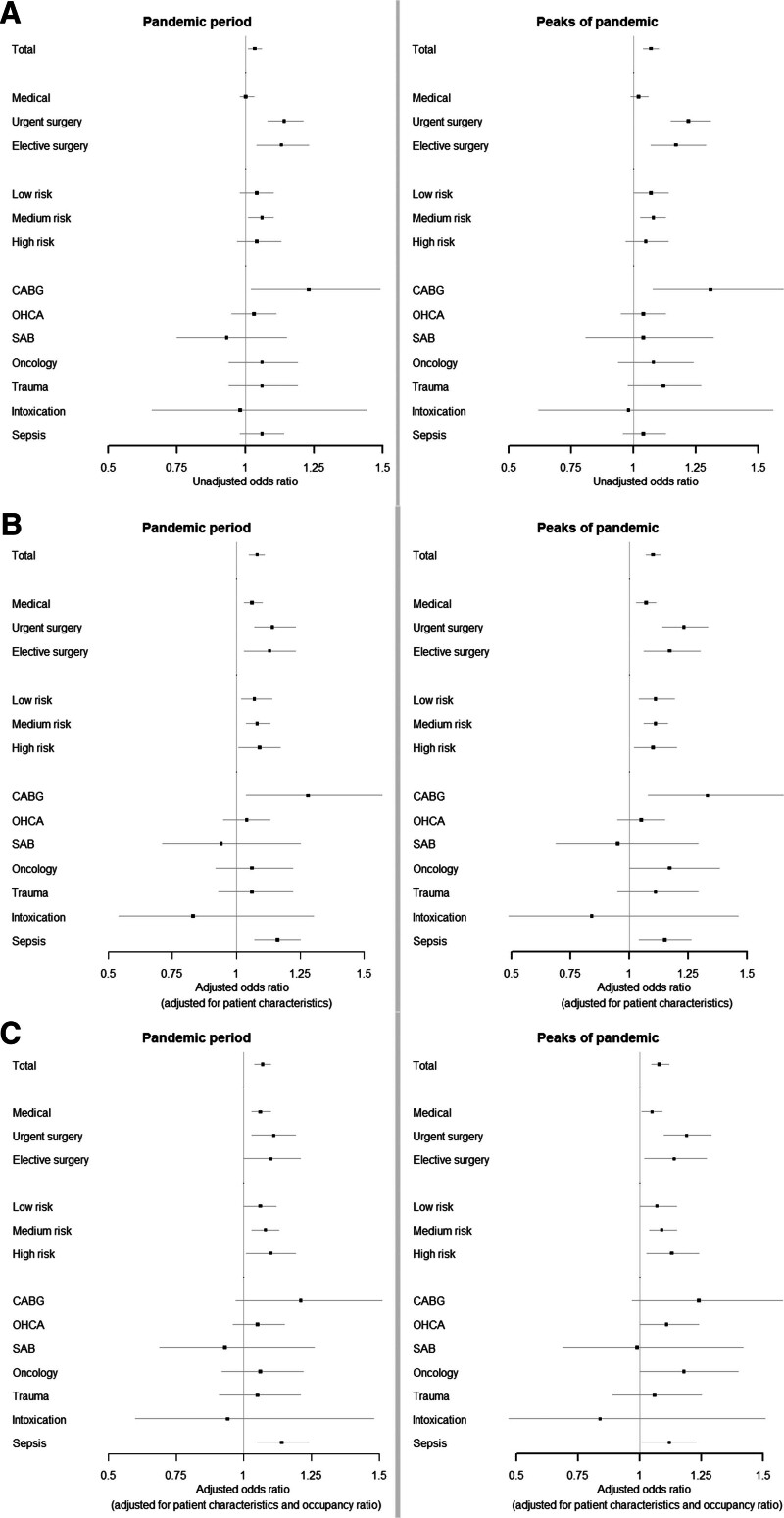
Influence of the pandemic years on the in-hospital mortality of non-COVID-19 patients expressed as odds ratios. **A**, Unadjusted. **B**, Adjusted for patient characteristics. **C**, Adjusted for patient characteristics and occupancy ratio. CABG = coronary artery bypass grafting, OHCA = out-of-hospital cardiac arrest, SAB = subarachnoid hemorrhage.

We had complete data on most model variables, there were 281 patients (0.1%) with missing age and 35,919 patients (12.6%) with missing BMI. These patients were included as separate category during the analyses. Both the unadjusted and for patient characteristic adjusted mixed-effects logistic regression models, showed that the in-hospital mortality for the non-COVID-19 ICU patients was slightly higher during the pandemic period and during its peaks compared with the prepandemic cohort [OR pandemic period: unadjusted 1.03 (95% CI, 1.01–1.06)] and adjusted 1.08 (95% CI, 1.05–1.11); OR peaks of pandemic: unadjusted 1.07 (95% CI, 1.04–1.10) and adjusted 1.10 (95% CI, 1.07–1.13) (**Fig. 3**).

For the admission type strata, both the unadjusted and adjusted models showed a higher in-hospital mortality for the non-COVID-19 ICU patients during the pandemic period and its peaks (with exception of the unadjusted OR of the medical patients in the pandemic period). For disease strata, unadjusted results showed a higher mortality for CABG patients during the pandemic and its peaks, whereas the adjusted results showed a higher mortality for CABG and sepsis patients during the pandemic period, and a higher mortality for CABG, oncology, and sepsis patients during the peaks of the pandemic. Adjusted results for the APACHE-IV mortality risk strata showed higher mortality in each risk stratum during the pandemic period and its peaks (Fig. 3).

**Figure 4.**
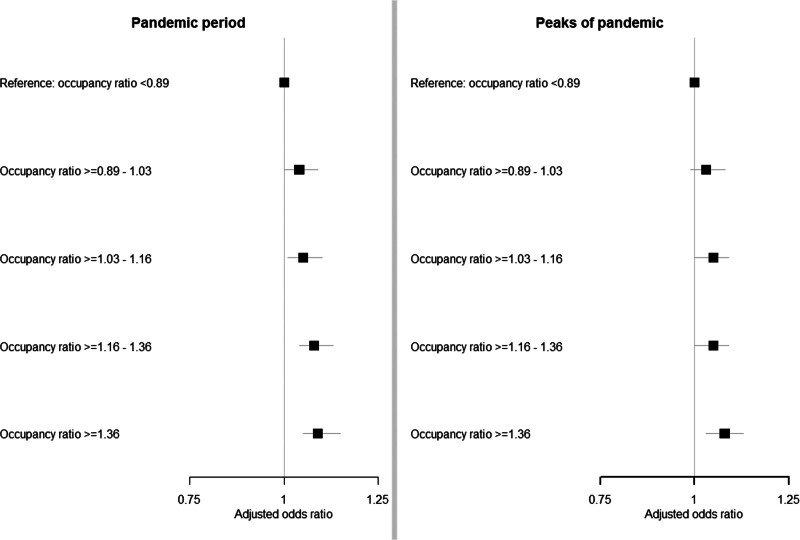
Influence of the occupancy ratio on the in-hospital mortality of non-COVID-19 patient expressed as the adjusted odds ratio.

When we added the quintiles of occupancy ratio at ICU admission to the adjusted model, the results remained similar. For the disease strata, the effect estimates remained similar, although only the sepsis patients showed higher in-hospital mortality (Fig. 3***C***). The associations between the quintiles of occupancy ratio itself and in-hospital mortality showed that, with the lowest occupancy ratio quintile as reference, higher occupancy ratio quintiles were associated with higher in-hospital mortality with an OR of 1.09 (95% CI, 1.05–1.15) for an occupancy ratio of 1.36 or higher (**Fig. 4**). This was similar for disease and severity strata (**Table S14**, http://links.lww.com/CCM/H469).

## DISCUSSION

This study indicates that strain on the ICU capacity has a considerable impact on the intensive care of a resource-optimized healthcare system. During the pandemic, there was a substantial decrease in non-COVID-19 ICU patient volume, especially during peak weeks, suggesting a need for stricter patient selection for ICU admission. The comorbidities of the admitted non-COVID-19 patients during the pandemic were lower compared with the prepandemic cohort. Therefore, we would expect lower mortality in the non-COVID-19 pandemic cohort. In contrast, the in-hospital mortality was modestly higher for the non-COVID-19 pandemic cohort, indicating that the effect size was underestimated. This is further substantiated by the observation that the adjusted OR exceeds the unadjusted OR in specific subgroups. The occupancy ratio was also associated with higher in-hospital mortality, indicating that factors, such as patient selection, higher workload, and higher stress levels, contributed to the modest increased in-hospital mortality. These present findings expand previous research that did not examine the entire two-year pandemic period and its peak weeks (18–21), only included regional data (6, 19, 20, 22), and did not stratify the results by disease or severity strata (6, 19–21, 23–26).

Notably, different disease categories showed varying effects, suggesting that the pandemic and government restrictions influenced healthcare mechanisms differently. For instance, there was less traffic because of the advice to work at home, reducing the risks of road accidents. Furthermore, fewer social contacts and more social distancing most likely reduced the spread of other infectious diseases as well. In addition, delays in elective surgery to create ICU capacity (3) might have resulted in more severely ill elective patients once they arrived at the ICU. This mechanism could explain the lower volumes and higher in-hospital mortality found in elective surgery and CABG patients admitted during the pandemic period. Of note is that the CABG patients in this study consisted mostly of elective surgery patients (93.1%). McAlister et al (27) also observed different outcomes among various non-COVID-19 subgroups, observing increased mortality in the subgroups of heart failure, chronic obstructive pulmonary disease, and asthma, while observing no increase in mortality in the subgroups of acute coronary syndrome and stroke when comparing the prepandemic period with the pandemic period.

During the pandemic in the Netherlands, the strain on ICU capacity was classified into three categories: normal, unforeseen, and crisis peak load. Especially during the crisis peak load, ICUs were forced to implement a stricter triage policy to optimize the scarce availability of ICU beds (28). Taken together, the selection of patients admitted to the ICU during the pandemic was present for each disease severity category, at least during the peaks of the pandemic. This would mean that higher case-mix adjusted in-hospital mortality cannot be explained by the presence of the most severely affected patients only, which is in line with the higher in-hospital mortality found in all APACHE-IV mortality risk strata.

This study has strengths and limitations. A limitation is that our results may not be generalizable to other country settings as we included the data of the Netherlands only, which has a well-equipped healthcare system, abundant resources, and low number of prepandemic ICU beds per capita. Importantly, declines in ICU treatment for non-COVID-19 disease (29) and other health services due to the pandemic also have struck other countries with fewer resources, likely affecting many more patients in a harmful way (21, 30, 31). Furthermore, patients that did not reach the ICU due to the stricter patient selection during the pandemic could not be included in this study. It is very likely that the COVID-19 pandemic has affected their morbidity and mortality as well. Finally, the exact number of surge beds per hospital per week was unclear and may have affected the results if surge bed numbers were modified during the pandemic. As areas in the Netherlands became pandemic hotspots sequentially, numbers of surge beds might have changed between hospitals over time.

Importantly, we included data of all ICU patients of an entire healthcare system over a 4-year study period. Thereby, we were able to expand previous findings on the impact of the COVID-19 pandemic on non-COVID-19 ICU patients (6, 8, 18–21, 25, 26). In particular, the Dutch setting with scarce ICU beds and the pandemic provided an opportunity to study what happened to ICU patients in a high-level and efficient healthcare system when strain on scarce ICU beds occurred. The results provide evidence that strain threatens the performance of such a healthcare system. Efficient high-level healthcare requires optimization of resources, while also taking scenarios of strain on scarce ICU beds into account, in order to do the most for the most (32, 33). In our study, we employed the occupancy ratio on the day of ICU admission as an indicator of the ICU’s strain level. Nevertheless, a more exact delineation of “strain” would be advantageous, given the current disparity in definitions across studies (27, 33–38). Disparities in definition may, to some extent, account for variations in the observed effect sizes regarding strain’s impact on mortality. This warrants further investigation.

## CONCLUSIONS

The COVID-19 pandemic has had considerable impact on Dutch ICU non-COVID-19 care, leading to reduced patient volumes for certain patient types, a different selection of patients, and a modest increase in case-mix adjusted in-hospital mortality, especially during the pandemic peaks when the strain on ICUs was extreme. This highlights the need for highly efficient healthcare systems, like the Dutch system, to reconsider prepandemic strategies and adopt best practices in critical care surge management to safeguard lives during unexpected surges in ICU admissions.

## Supplementary Material


