# Supplementary material for: Strain on Scarce Intensive Care Beds Drives Reduced Patient Volumes, Patient Selection, and Worse Outcome: A National Cohort Study*
Source: Crit Care Med. 2023 Dec 14;52(4):574–85. doi: 10.1097/CCM.0000000000006156 (PMC10930373; doi:10.1097/CCM.0000000000006156)
Supplement: Supplementary file 1 [file ccm-52-0574-s001.docx]

**Appendix**

Content

[Figure S1: Number of occupied ICU beds 2](#_Toc146533233)

[Figure S2: Flow diagram of included non-COVID-19 patients 3](#_Toc146533234)

[Table S1: Demographics of the medical non-COVID-19 patients 4](#_Toc146533235)

[Table S2: Demographics of the urgent surgery non-COVID-19 patients 5](#_Toc146533236)

[Table S3: Demographics of the elective surgery non-COVID-19 patients 6](#_Toc146533237)

[Table S4: Demographics of the CABG non-COVID-19 patients 7](#_Toc146533238)

[Table S5: Demographics of the OHCA non-COVID-19 patients 8](#_Toc146533239)

[Table S6: Demographics of the SAB non-COVID-19 patients 9](#_Toc146533240)

[Table S7: Demographics of the oncology non-COVID-19 patients 10](#_Toc146533241)

[Table S8: Demographics of the trauma non-COVID-19 patients 11](#_Toc146533242)

[Table S9: Demographics of the intoxication non-COVID-19 patients 12](#_Toc146533243)

[Table S10: Demographics of the sepsis non-COVID-19 patients 13](#_Toc146533244)

[Table S11: Demographics of the low APACHE IV risk (<20%) non-COVID-19 patients 14](#_Toc146533245)

[Table S12: Demographics of the medium APACHE IV risk (20-70%) non-COVID-19 patients 15](#_Toc146533246)

[Table S13: Demographics of the high APACHE IV risk (≥70%) non-COVID-19 patients 16](#_Toc146533247)

[Table S14: Influence of the occupancy rate on the in-hospital mortality in subgroups of the non-COVID-19 patient expressed as the odds ratio 17](#_Toc146533248)


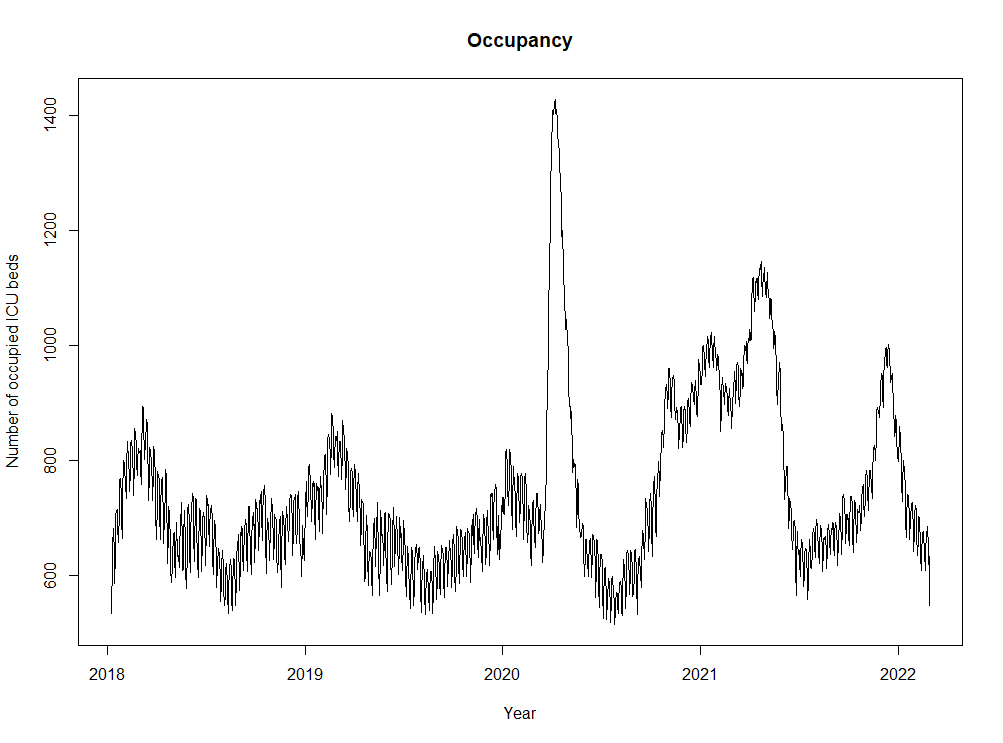


Figure S1: Number of occupied ICU beds

Dutch ICU patients studied

N=285,130

Historic control Dutch ICU patients cohort

January 1^st^ 2018 – December 31^st^ 2019

N=164,737

Non-COVID-19 Dutch ICU patients cohort

March 1^st^ 2020 – February 28^st^ 2022

N=120,393

Historic control Dutch ICU patients cohort for matched peak weeks of the pandemic

N=109,741

Non-COVID-19 Dutch ICU patients cohort

in peak weeks of pandemic

N=72,173

Figure S2: Flow diagram of included non-COVID-19 patients

# Table S1: Demographics of the medical non-COVID-19 patients

|  | **Pandemic period: reference** | **Pandemic period:**  **non-COVID** | **Peaks of pandemic: reference** | **Peaks of pandemic: non-COVID** |
| --- | --- | --- | --- | --- |
| Number of patients | 83445 | 57952 | 55897.5 | 34882 |
| Age, median (IQR) | 65 (52-74) | 64 (50-73) | 66 (53-75) | 64 (51-73) |
| Male (%) | 57.9 | 59 | 57.7 | 59.3 |
| Comorbidities (%) |  |  |  |  |
| Malignancy | 7.5 | 6.2 | 7.4 | 6.1 |
| Immunological | 11.6 | 9.1 | 11.5 | 9.1 |
| Respiratory insufficiency | 19.9 | 14 | 20.1 | 13.6 |
| Renal insufficiency | 8.6 | 6.2 | 8.6 | 6.1 |
| Cardiovasculair insufficiency | 4.7 | 3.7 | 4.7 | 3.6 |
| Liver cirrhosis | 3 | 2.1 | 3 | 2.1 |
| Diabetes mellitus | 18.7 | 17.3 | 18.6 | 17.3 |
| Supportive treatment (%) |  |  |  |  |
| Mechanically ventilated in  1st 24hrs of ICU admission | 39.5 | 39.2 | 40 | 40.9 |
| Vasoactive medication | 34.8 | 39.2 | 35.1 | 41 |
| Disease severity scores median (IQR) |  |  |  |  |
| APACHE III APS | 45 (28-67) | 46 (29-70) | 46 (29-67) | 47 (30-71) |
| APACHE III score | 59 (39-82) | 59 (39-83) | 59 (39-82) | 60 (39-84) |
| Hospital mortality (%) | 15427 (18.5) | 10943 (18.9) | 20983 (18.8) | 6821 (19.6) |
| Length of ICU stay, median (IQR) | 1.6 (0.7-3.7) | 1.4 (0.7-3.2) | 1.6 (0.7-3.7) | 1.4 (0.7-3.2) |
| Length of hospital stay, median (IQR) | 8 (3-16.4) | 7 (3-15.5) | 8 (3-16.5) | 7 (3-16) |

# Table S2: Demographics of the urgent surgery non-COVID-19 patients

|  | **Pandemic period: reference** | **Pandemic period: non-COVID** | **Peaks of pandemic: reference** | **Peaks of pandemic: non-COVID** |
| --- | --- | --- | --- | --- |
| Number of patients | 18941 | 14802 | 12583 | 9210 |
| Age, median (IQR) | 66 (51-75) | 66 (53-75) | 66 (52-75) | 66 (54-75) |
| Male (%) | 57.4 | 59.3 | 57.5 | 59.7 |
| Comorbidities (%) |  |  |  |  |
| Malignancy | 5.8 | 4.9 | 5.8 | 5 |
| Immunological | 7.9 | 6.4 | 8 | 6.6 |
| Respiratory insufficiency | 11.8 | 10.2 | 11.9 | 10.3 |
| Renal insufficiency | 6.8 | 5 | 6.9 | 5 |
| Cardiovasculair insufficiency | 5.7 | 6 | 5.8 | 6.4 |
| Liver cirrhosis | 2.3 | 1.3 | 2.3 | 1.4 |
| Diabetes mellitus | 13.3 | 13.2 | 13.4 | 13.2 |
| Supportive treatment (%) |  |  |  |  |
| Mechanically ventilated in  1st 24hrs of ICU admission | 55.8 | 63.3 | 55.9 | 64.6 |
| Vasoactive medication | 52.7 | 63.3 | 52.9 | 64.8 |
| Disease severity scores median (IQR) |  |  |  |  |
| APACHE III APS | 41 (25-59) | 44 (30-62) | 41 (25-60) | 44 (30-63) |
| APACHE III score | 54 (35-74) | 57 (40-76) | 54 (35-74) | 57 (40-77) |
| Hospital mortality (%) | 2752 (14.5) | 2473 (16.7) | 3631 (14.4) | 1651 (17.9) |
| Length of ICU stay, median (IQR) | 1.3 (0.6-3.6) | 1.4 (0.7-3.6) | 1.4 (0.6-3.7) | 1.4 (0.7-3.5) |
| Length of hospital stay, median (IQR) | 11 (5-21.6) | 11 (5.5-21.5) | 11 (5-21.6) | 11 (5.6-21.7) |

# Table S3: Demographics of the elective surgery non-COVID-19 patients

|  | **Pandemic period: reference** | **Pandemic period: non-COVID** | **Peaks of pandemic: reference** | **Peaks of pandemic: non-COVID** |
| --- | --- | --- | --- | --- |
| Number of patients | 53098 | 39183 | 35106 | 23471 |
| Age, median (IQR) | 67 (58-74) | 68 (59-74) | 68 (58-74) | 68 (60-74) |
| Male (%) | 64.9 | 67.5 | 65 | 67.9 |
| Comorbidities (%) |  |  |  |  |
| Malignancy | 6.1 | 6.1 | 6 | 6.3 |
| Immunological | 7.8 | 7.5 | 7.8 | 7.6 |
| Respiratory insufficiency | 13.3 | 10.8 | 13.1 | 10.3 |
| Renal insufficiency | 4.5 | 3.5 | 4.5 | 3.3 |
| Cardiovasculair insufficiency | 4.8 | 4.2 | 4.8 | 4.1 |
| Liver cirrhosis | 0.7 | 0.4 | 0.6 | 0.3 |
| Diabetes mellitus | 16.6 | 16.9 | 16.5 | 16.8 |
| Supportive treatment (%) |  |  |  |  |
| Mechanically ventilated in  1st 24hrs of ICU admission | 49.2 | 57.1 | 49.1 | 58.4 |
| Vasoactive medication | 45.6 | 55.3 | 45.6 | 56.5 |
| Disease severity scores median (IQR) |  |  |  |  |
| APACHE III APS | 30 (22-40) | 31 (23-41) | 30 (22-40) | 31 (23-41) |
| APACHE III score | 43 (32-54) | 44 (34-55) | 43 (32-54) | 44 (34-55) |
| Hospital mortality (%) | 1253 (2.4) | 1035 (2.6) | 1637 (2.3) | 636 (2.7) |
| Length of ICU stay, median (IQR) | 0.9 (0.8-1.1) | 0.9 (0.8-1) | 0.9 (0.8-1.1) | 0.9 (0.7-1) |
| Length of hospital stay, median (IQR) | 7.4 (5-12) | 7 (5-11.6) | 7.5 (5-12) | 7.5 (5-12) |

#

# Table S4: Demographics of the CABG non-COVID-19 patients

|  | **Pandemic period: reference** | **Pandemic period: non-COVID** | **Peaks of pandemic: reference** | **Peaks of pandemic: non-COVID** |
| --- | --- | --- | --- | --- |
| Number of patients | 15303 | 14227 | 10150 | 8906 |
| Age, median (IQR) | 69 (61-74) | 68 (61-74) | 69 (61-74) | 69 (61-74) |
| Male (%) | 80.7 | 80.8 | 80.7 | 80.4 |
| Admission type (%) |  |  |  |  |
| Medical admission | 0 | 0 | 0 | 0 |
| Urgent admission | 7.0 | 9.6 | 7.0 | 10.0 |
| Elective admission | 93.0 | 90.4 | 93.0 | 90.0 |
| Comorbidities (%) |  |  |  |  |
| Malignancy | 0.7 | 0.8 | 0.7 | 0.7 |
| Immunological | 2.7 | 2.6 | 2.8 | 2.6 |
| Respiratory insufficiency | 8.4 | 6.6 | 8.2 | 6.4 |
| Renal insufficiency | 4 | 2.8 | 3.9 | 2.8 |
| Cardiovasculair insufficiency | 9.9 | 8 | 10.2 | 7.9 |
| Liver cirrhosis | 0.2 | 0.2 | 0.2 | 0.2 |
| Diabetes mellitus | 22.4 | 23.8 | 22.5 | 23.4 |
| Supportive treatment (%) |  |  |  |  |
| Mechanically ventilated in  1st 24hrs of ICU admission | 95.8 | 95.3 | 95.5 | 95 |
| Vasoactive medication | 69.4 | 71.5 | 69.7 | 71.6 |
| Disease severity scores median (IQR) |  |  |  |  |
| APACHE III APS | 33 (24-42) | 32 (24-42) | 33 (24-42) | 32 (24-41) |
| APACHE III score | 45 (36-56) | 45 (36-56) | 45 (36-56) | 45 (36-55) |
| Hospital mortality (%) | 206 (1.3) | 240 (1.7) | 279 (1.4) | 166 (1.9) |
| Length of ICU stay, median (IQR) | 0.9 (0.7-1.1) | 0.9 (0.7-1) | 0.9 (0.7-1.1) | 0.9 (0.7-1) |
| Length of hospital stay, median (IQR) | 7 (5.6-10.6) | 7 (5.5-10) | 7.5 (5.6-11) | 7 (5.5-10.5) |

# Table S5: Demographics of the OHCA non-COVID-19 patients

|  | **Pandemic period: reference** | **Pandemic period: non-COVID** | **Peaks of pandemic: reference** | **Peaks of pandemic: non-COVID** |
| --- | --- | --- | --- | --- |
| Number of patients | 5601 | 4720 | 3762.5 | 3050 |
| Age, median (IQR) | 67 (55-75) | 65 (54-73) | 67 (55-75) | 65 (55-73) |
| Male (%) | 70.4 | 71.2 | 70.5 | 71.2 |
| Admission type (%) |  |  |  |  |
| Medical admission | 98.7 | 98.7 | 98.7 | 98.7 |
| Urgent admission | 0.7 | 0.7 | 0.8 | 0.7 |
| Elective admission | 0.1 | 0 | 0.1 | 0.1 |
| Comorbidities (%) |  |  |  |  |
| Malignancy | 14.3 | 2.6 | 13.3 | 2.7 |
| Immunological | 15.6 | 3.7 | 14.8 | 3.9 |
| Respiratory insufficiency | 23.4 | 10.4 | 23.1 | 10.2 |
| Renal insufficiency | 16.3 | 4.4 | 15.2 | 4.4 |
| Cardiovasculair insufficiency | 16.1 | 5.1 | 15.1 | 5.2 |
| Liver cirrhosis | 12.4 | 0.9 | 11.3 | 0.9 |
| Diabetes mellitus | 25.5 | 13.9 | 24.6 | 14.7 |
| Supportive treatment (%) |  |  |  |  |
| Mechanically ventilated in  1st 24hrs of ICU admission | 94.1 | 93.5 | 94.1 | 93.7 |
| Vasoactive medication | 71.8 | 84.3 | 72.9 | 84.5 |
| Disease severity scores median (IQR) |  |  |  |  |
| APACHE III APS | 90 (59-111) | 92 (71-111) | 90 (60-111) | 93 (72-112) |
| APACHE III score | 101 (70-124) | 103 (82-123) | 101 (72-124) | 104 (82-124) |
| Hospital mortality (%) | 2433 (43.4) | 2279 (48.3) | 3325 (44.2) | 1500 (49.2) |
| Length of ICU stay, median (IQR) | 2 (0.7-4.3) | 2.2 (0.9-4.6) | 2.1 (0.7-4.5) | 2.1 (0.8-4.3) |
| Length of hospital stay, median (IQR) | 5 (2-13) | 5 (2-13) | 5 (2-13) | 5 (1.9-13) |

# Table S6: Demographics of the SAB non-COVID-19 patients

|  | **Pandemic period: reference** | **Pandemic period: non-COVID** | **Peaks of pandemic: reference** | **Peaks of pandemic: non-COVID** |
| --- | --- | --- | --- | --- |
| Number of patients | 997 | 881 | 656.5 | 544 |
| Age, median (IQR) | 59 (51-68) | 59 (49-68) | 60 (51-68) | 60 (50-68) |
| Male (%) | 37.3 | 36.5 | 37.7 | 36.6 |
| Admission type (%) |  |  |  |  |
| Medical admission | 98 | 99 | 98.1 | 98.9 |
| Urgent admission | 1 | 0.1 | 0.8 | 0 |
| Elective admission | 0.2 | 0 | 0.1 | 0 |
| Comorbidities (%) |  |  |  |  |
| Malignancy | 1.3 | 0.9 | 1.1 | 0.9 |
| Immunological | 2 | 1.7 | 2 | 2 |
| Respiratory insufficiency | 6 | 4.4 | 5.9 | 4.4 |
| Renal insufficiency | 1.3 | 0.5 | 1.1 | 0.6 |
| Cardiovasculair insufficiency | 1.2 | 0.6 | 1.4 | 0.6 |
| Liver cirrhosis | 0.3 | 0.2 | 0.3 | 0.4 |
| Diabetes mellitus | 4.8 | 4.5 | 5.2 | 5.5 |
| Supportive treatment (%) |  |  |  |  |
| Mechanically ventilated in  1st 24hrs of ICU admission | 46.1 | 47.7 | 45.4 | 49.4 |
| Vasoactive medication | 38.5 | 49.6 | 38 | 51.8 |
| Disease severity scores median (IQR) |  |  |  |  |
| APACHE III APS | 39 (24-73) | 41 (25-70) | 38 (24-73) | 42 (25.8-72) |
| APACHE III score | 50 (32-83) | 51 (33-80) | 49 (32-83) | 53 (34-83) |
| Hospital mortality (%) | 267 (26.8) | 239 (27.1) | 346 (26.4) | 157 (28.9) |
| Length of ICU stay, median (IQR) | 2.2 (1-5.2) | 2.5 (1-5.6) | 2.1 (0.9-5.1) | 2.5 (1-5.7) |
| Length of hospital stay, median (IQR) | 12 (3-21.5) | 12.5 (4-22) | 12 (3-21) | 12.5 (3.6-21.8) |

# Table S7: Demographics of the oncology non-COVID-19 patients

|  | **Pandemic period: reference** | **Pandemic period: non-COVID** | **Peaks of pandemic: reference** | **Peaks of pandemic: non-COVID** |
| --- | --- | --- | --- | --- |
| Number of patients | 12326 | 8585 | 8088 | 5272 |
| Age, median (IQR) | 67 (58-73) | 67 (59-74) | 67 (58-73) | 67 (59-74) |
| Male (%) | 58.1 | 56.8 | 58.1 | 57 |
| Admission type (%) |  |  |  |  |
| Medical admission | 9.7 | 9.5 | 10 | 8.7 |
| Urgent admission | 3.9 | 4 | 3.8 | 4.1 |
| Elective admission | 86.4 | 86.3 | 86.2 | 87 |
| Comorbidities (%) |  |  |  |  |
| Malignancy | 25.1 | 26.7 | 25.2 | 26.8 |
| Immunological | 23.1 | 23.8 | 23.1 | 23.9 |
| Respiratory insufficiency | 14.9 | 13.4 | 14.9 | 13.1 |
| Renal insufficiency | 2.7 | 2.4 | 2.7 | 2.4 |
| Cardiovasculair insufficiency | 1.5 | 1.4 | 1.5 | 1.2 |
| Liver cirrhosis | 0.4 | 0.5 | 0.5 | 0.5 |
| Diabetes mellitus | 13.3 | 12.7 | 13.2 | 12.5 |
| Supportive treatment (%) |  |  |  |  |
| Mechanically ventilated in  1st 24hrs of ICU admission | 19.6 | 20.3 | 19.7 | 19.9 |
| Vasoactive medication | 34.7 | 40 | 34.8 | 40.5 |
| Disease severity scores median (IQR) |  |  |  |  |
| APACHE III APS | 32 (24-42) | 32 (25-41) | 32 (25-42) | 32 (24-41) |
| APACHE III score | 45 (35-56) | 45 (35-56) | 45 (35-56) | 45 (35-56) |
| Hospital mortality (%) | 735 (6) | 517 (6) | 958 (5.9) | 312 (5.9) |
| Length of ICU stay, median (IQR) | 0.9 (0.8-1.7) | 0.9 (0.8-1.1) | 0.9 (0.8-1.7) | 0.9 (0.8-1.1) |
| Length of hospital stay, median (IQR) | 8.8 (6-14.4) | 8.6 (5.7-14) | 9 (6-14.5) | 8.6 (5.9-14) |

# Table S8: Demographics of the trauma non-COVID-19 patients

|  | **Pandemic period: reference** | **Pandemic period: non-COVID** | **Peaks of pandemic: reference** | **Peaks of pandemic: non-COVID** |
| --- | --- | --- | --- | --- |
| Number of patients | 7251 | 5393 | 4631.5 | 3045 |
| Age, median (IQR) | 59 (39-75) | 58 (37-73) | 59 (39-76) | 58 (38-73) |
| Male (%) | 64.9 | 69.7 | 64.3 | 69.2 |
| Admission type (%) |  |  |  |  |
| Medical admission | 60 | 65 | 59.5 | 64.7 |
| Urgent admission | 22.6 | 26.2 | 23 | 27.1 |
| Elective admission | 17.2 | 8.4 | 17.4 | 7.7 |
| Comorbidities (%) |  |  |  |  |
| Malignancy | 1.2 | 1.4 | 1.3 | 1.2 |
| Immunological | 2.4 | 2.1 | 2.4 | 2.1 |
| Respiratory insufficiency | 7.7 | 6.4 | 7.8 | 6.6 |
| Renal insufficiency | 3 | 2.2 | 3.2 | 2.1 |
| Cardiovasculair insufficiency | 1.7 | 1.4 | 1.7 | 1.3 |
| Liver cirrhosis | 0.5 | 0.5 | 0.4 | 0.5 |
| Diabetes mellitus | 8.8 | 7.7 | 9.2 | 7.9 |
| Supportive treatment (%) |  |  |  |  |
| Mechanically ventilated in  1st 24hrs of ICU admission | 33.8 | 38.7 | 33.7 | 41.3 |
| Vasoactive medication | 28.5 | 35 | 29.1 | 37.1 |
| Disease severity scores median (IQR) |  |  |  |  |
| APACHE III APS | 32 (22-48) | 34 (23-51) | 32 (22-48) | 35 (24-53) |
| APACHE III score | 43 (28-62) | 44 (30-64) | 43 (28-62) | 45 (30-65) |
| Hospital mortality (%) | 737 (10.2) | 633 (11.7) | 962 (10.4) | 394 (12.9) |
| Length of ICU stay, median (IQR) | 1.1 (0.7-2.8) | 1 (0.7-2.8) | 1.1 (0.7-2.9) | 1 (0.6-2.8) |
| Length of hospital stay, median (IQR) | 8 (4-15) | 8 (4-15.6) | 8 (4-15.4) | 8 (4-16) |

# Table S9: Demographics of the intoxication non-COVID-19 patients

|  | **Pandemic period: reference** | **Pandemic period: non-COVID** | **Peaks of pandemic: reference** | **Peaks of pandemic: non-COVID** |
| --- | --- | --- | --- | --- |
| Number of patients | 6375 | 5090 | 4126.5 | 2973 |
| Age, median (IQR) | 40 (27-53) | 38 (26-52) | 39 (27-53) | 38 (26-53) |
| Male (%) | 39.3 | 36.7 | 39.4 | 36.9 |
| Admission type (%) |  |  |  |  |
| Medical admission | 99.8 | 99.9 | 99.8 | 99.8 |
| Urgent admission | 0 | 0 | 0 | 0 |
| Elective admission | 0 | 0 | 0 | 0 |
| Comorbidities (%) |  |  |  |  |
| Malignancy | 0.6 | 0.7 | 0.5 | 0.8 |
| Immunological | 1.2 | 1.2 | 1.1 | 1.1 |
| Respiratory insufficiency | 4.3 | 3.8 | 4.3 | 3.5 |
| Renal insufficiency | 0.7 | 0.7 | 0.7 | 0.7 |
| Cardiovasculair insufficiency | 0.2 | 0.1 | 0.2 | 0.1 |
| Liver cirrhosis | 0.3 | 0.2 | 0.3 | 0.2 |
| Diabetes mellitus | 5.1 | 5.2 | 5.1 | 5.4 |
| Supportive treatment (%) |  |  |  |  |
| Mechanically ventilated in  1st 24hrs of ICU admission | 13.9 | 14.2 | 14.3 | 15.3 |
| Vasoactive medication | 6.3 | 7.7 | 6.6 | 9.1 |
| Disease severity scores median (IQR) |  |  |  |  |
| APACHE III APS | 30 (21-46) | 31 (20-47) | 30 (21-47) | 31 (20-49) |
| APACHE III score | 34 (23-51) | 34 (23-52) | 34 (23-52) | 34 (23-53) |
| Hospital mortality (%) | 58 (0.9) | 46 (0.9) | 70 (0.8) | 26 (0.9) |
| Length of ICU stay, median (IQR) | 0.7 (0.5-1.1) | 0.7 (0.5-1) | 0.7 (0.5-1.1) | 0.7 (0.5-1) |
| Length of hospital stay, median (IQR) | 1.5 (1-2) | 1.5 (1-2.5) | 1.5 (1-2) | 1.5 (1-2.6) |

# Table S10: Demographics of the sepsis non-COVID-19 patients

|  | **Pandemic period: reference** | **Pandemic period: non-COVID** | **Peaks of pandemic: reference** | **Peaks of pandemic: non-COVID** |
| --- | --- | --- | --- | --- |
| Number of patients | 10038 | 6418 | 6622 | 3882 |
| Age, median (IQR) | 69 (59-77) | 69 (58-76) | 69 (59-77) | 68 (58-75) |
| Male (%) | 57.7 | 57.8 | 57.9 | 57.8 |
| Admission type (%) |  |  |  |  |
| Medical admission | 77.5 | 75.5 | 77.4 | 74.4 |
| Urgent admission | 18.8 | 21.4 | 18.8 | 22.6 |
| Elective admission | 3.7 | 3 | 3.6 | 2.9 |
| Comorbidities (%) |  |  |  |  |
| Malignancy | 12 | 12.8 | 11.6 | 12.7 |
| Immunological | 19.8 | 18.5 | 19.6 | 18.9 |
| Respiratory insufficiency | 16.8 | 13.1 | 16.8 | 12.4 |
| Renal insufficiency | 11.9 | 10.1 | 12.1 | 9.9 |
| Cardiovasculair insufficiency | 3.8 | 4 | 3.9 | 4.1 |
| Liver cirrhosis | 2.4 | 2.2 | 2.5 | 2.1 |
| Diabetes mellitus | 23.9 | 23 | 23.5 | 22.2 |
| Supportive treatment (%) |  |  |  |  |
| Mechanically ventilated in  1st 24hrs of ICU admission | 42.3 | 40.4 | 43.1 | 41.5 |
| Vasoactive medication | 73.8 | 81.1 | 73.9 | 83.2 |
| Disease severity scores median (IQR) |  |  |  |  |
| APACHE III APS | 61 (46-80) | 61 (47-79) | 61 (46-80) | 62 (47-80) |
| APACHE III score | 77 (60-97) | 77 (61-95) | 77 (61-97) | 77 (61-96) |
| Hospital mortality (%) | 2619 (26.1) | 1758 (27.4) | 3478 (26.3) | 1064 (27.4) |
| Length of ICU stay, median (IQR) | 2.4 (1.1-5.5) | 2 (1-4.5) | 2.4 (1.1-5.5) | 1.9 (0.9-4.4) |
| Length of hospital stay, median (IQR) | 12.6 (6-25) | 12 (6-24.5) | 13 (6-25.7) | 12 (6-25) |

# Table S11: Demographics of the low APACHE IV risk (<20%) non-COVID-19 patients

|  | **Pandemic period: reference** | **Pandemic period: non-COVID** | **Peaks of pandemic: reference** | **Peaks of pandemic: non-COVID** |
| --- | --- | --- | --- | --- |
| Number of patients | 99562 | 75904 | 65800.5 | 45201 |
| Age, median (IQR) | 64 (52-73) | 64 (53-73) | 65 (52-73) | 65 (53-73) |
| Male (%) | 60.6 | 62.2 | 60.6 | 62.6 |
| Admission type (%) |  |  |  |  |
| Medical admission | 42.5 | 40.6 | 42.7 | 40 |
| Urgent admission | 9.5 | 10.8 | 9.5 | 11.1 |
| Elective admission | 47.9 | 48.6 | 47.8 | 48.9 |
| Comorbidities (%) |  |  |  |  |
| Malignancy | 4.1 | 4.1 | 4.1 | 4.3 |
| Immunological | 6.8 | 6.2 | 6.8 | 6.2 |
| Respiratory insufficiency | 15.1 | 11.4 | 15.1 | 10.9 |
| Renal insufficiency | 4.5 | 3.7 | 4.6 | 3.5 |
| Cardiovasculair insufficiency | 3.8 | 3.7 | 3.9 | 3.7 |
| Liver cirrhosis | 0.8 | 0.7 | 0.8 | 0.7 |
| Diabetes mellitus | 16.2 | 15.9 | 16.2 | 15.9 |
| Supportive treatment (%) |  |  |  |  |
| Mechanically ventilated in  1st 24hrs of ICU admission | 39.5 | 43.3 | 39.7 | 44.7 |
| Vasoactive medication | 35.4 | 41.8 | 35.5 | 43.4 |
| Disease severity scores median (IQR) |  |  |  |  |
| APACHE III APS | 32 (23-42) | 32 (24-43) | 32 (24-43) | 33 (24-43) |
| APACHE III score | 44 (33-56) | 44 (33-56) | 44 (33-56) | 44 (34-56) |
| Hospital mortality (%) | 3001 (3) | 2359 (3.1) | 3942 (3) | 1446 (3.2) |
| Length of ICU stay, median (IQR) | 1 (0.8-1.9) | 0.9 (0.7-1.7) | 1 (0.8-1.9) | 0.9 (0.7-1.7) |
| Length of hospital stay, median (IQR) | 7 (4-12) | 7 (4-11.8) | 7 (4-12.3) | 7 (4.1-12) |

# Table S12: Demographics of the medium APACHE IV risk (20-70%) non-COVID-19 patients

|  | **Pandemic period: reference** | **Pandemic period: non-COVID** | **Peaks of pandemic: reference** | **Peaks of pandemic: non-COVID** |
| --- | --- | --- | --- | --- |
| Number of patients | 27519 | 19539 | 18624.5 | 12033 |
| Age, median (IQR) | 71 (61-78) | 69 (59-76) | 71 (61-78) | 69 (59-76) |
| Male (%) | 59.5 | 61.7 | 59.5 | 61.8 |
| Admission type (%) |  |  |  |  |
| Medical admission | 79.4 | 76 | 79.6 | 75.5 |
| Urgent admission | 16 | 19.6 | 15.8 | 20.3 |
| Elective admission | 4.6 | 4.4 | 4.5 | 4.2 |
| Comorbidities (%) |  |  |  |  |
| Malignancy | 12.1 | 10.9 | 11.8 | 10.3 |
| Immunological | 16.3 | 13.8 | 16.2 | 13.8 |
| Respiratory insufficiency | 22.1 | 15.8 | 22.5 | 15.5 |
| Renal insufficiency | 12 | 9.2 | 11.9 | 9 |
| Cardiovasculair insufficiency | 5.4 | 5.1 | 5.5 | 5.1 |
| Liver cirrhosis | 3.1 | 3.2 | 3.1 | 3.1 |
| Diabetes mellitus | 21.4 | 19.8 | 21 | 19.5 |
| Supportive treatment (%) |  |  |  |  |
| Mechanically ventilated in  1st 24hrs of ICU admission | 61.3 | 63.6 | 61.4 | 65.1 |
| Vasoactive medication | 60.1 | 67.1 | 59.9 | 68.5 |
| Disease severity scores median (IQR) |  |  |  |  |
| APACHE III APS | 66 (55-79) | 68 (56-81) | 66 (54-80) | 68 (56-81) |
| APACHE III score | 82 (72-94) | 83 (72-95) | 82 (72-94) | 83 (72-95) |
| Hospital mortality (%) | 8349 (30.3) | 6183 (31.6) | 11330 (30.4) | 3884 (32.3) |
| Length of ICU stay, median (IQR) | 2.9 (1.4-6.6) | 2.6 (1.1-5.9) | 2.9 (1.4-6.6) | 2.5 (1.1-5.9) |
| Length of hospital stay, median (IQR) | 12.4 (6-22.9) | 12 (5.5-22) | 12.4 (6-23) | 12 (5.4-22.5) |

# Table S13: Demographics of the high APACHE IV risk (≥70%) non-COVID-19 patients

|  | **Pandemic period: reference** | **Pandemic period: non-COVID** | **Peaks of pandemic: reference** | **Peaks of pandemic: non-COVID** |
| --- | --- | --- | --- | --- |
| Number of patients | 7477 | 5600 | 5035 | 3555 |
| Age, median (IQR) | 71 (63-78) | 70 (61-76) | 71 (63-78) | 70 (61-76) |
| Male (%) | 62.8 | 64.4 | 62.4 | 65.1 |
| Admission type (%) |  |  |  |  |
| Medical admission | 89.2 | 89.9 | 89.3 | 89.1 |
| Urgent admission | 9.9 | 9.5 | 9.7 | 10.3 |
| Elective admission | 0.9 | 0.6 | 1 | 0.6 |
| Comorbidities (%) |  |  |  |  |
| Malignancy | 14.6 | 13.7 | 14 | 12.9 |
| Immunological | 16.4 | 14.2 | 16.1 | 13.5 |
| Respiratory insufficiency | 18.5 | 13.4 | 18.9 | 13.4 |
| Renal insufficiency | 10.5 | 8.4 | 10.9 | 8.2 |
| Cardiovasculair insufficiency | 5.5 | 5.6 | 5.6 | 5.2 |
| Liver cirrhosis | 4.5 | 4.1 | 4.7 | 3.9 |
| Diabetes mellitus | 21 | 19.8 | 21.1 | 20 |
| Supportive treatment (%) |  |  |  |  |
| Mechanically ventilated in  1st 24hrs of ICU admission | 89 | 89.6 | 88.8 | 90.2 |
| Vasoactive medication | 83.3 | 87 | 83.5 | 87 |
| Disease severity scores median (IQR) |  |  |  |  |
| APACHE III APS | 109 (95-125) | 109 (96-124) | 109 (95-125) | 109 (96-125) |
| APACHE III score | 125 (111-140) | 124 (112-139) | 125 (111-140) | 124 (112-139) |
| Hospital mortality (%) | 5027 (67.2) | 3813 (68.1) | 6769 (67.2) | 2423 (68.2) |
| Length of ICU stay, median (IQR) | 2.5 (0.9-6.1) | 2.2 (0.9-5.5) | 2.6 (0.9-6.2) | 2.1 (0.9-5.2) |
| Length of hospital stay, median (IQR) | 6 (2-17) | 5.2 (2-15.7) | 6 (2-16.6) | 5.1 (2-16) |

# Table S14: Influence of the occupancy rate on the in-hospital mortality in subgroups of the non-COVID-19 patient expressed as the odds ratio (abstracted from the model adjusted for patient characteristics and occupancy rate)

|  | **Odds ratio (95% CI) pandemic period** | **Odds ratio (95% CI)**  **peaks of pandemic** |
| --- | --- | --- |
| **Total** |  |  |
| Reference: occupancy.rate<0.89 | 1 (1-1) | 1 (1-1) |
| Occupancy rate >=0.89 - <1.03 | 1.04 (1.00-1.09) | 1.03 (0.99-1.08) |
| Occupancy rate >=1.03 - <1.16 | 1.05 (1.01-1.10) | 1.05 (1.00-1.09) |
| Occupancy rate >=1.16 - <1.36 | 1.08 (1.04-1.13) | 1.05 (1.00-1.09) |
| Occupancy rate >=1.36 | 1.09 (1.05-1.15) | 1.08 (1.03-1.13) |
| **Medical** |  |  |
| Reference: occupancy.rate<0.89 | 1 (1-1) | 1 (1-1) |
| Occupancy rate >=0.89 - <1.03 | 1.06 (1.01-1.11) | 1.05 (1.00-1.11) |
| Occupancy rate >=1.03 - <1.16 | 1.07 (1.02-1.13) | 1.07 (1.02-1.12) |
| Occupancy rate >=1.16 - <1.36 | 1.09 (1.04-1.15) | 1.07 (1.02-1.12) |
| Occupancy rate >=1.36 | 1.08 (1.02-1.14) | 1.08 (1.03-1.14) |
| **Urgent surgery** |  |  |
| Reference: occupancy.rate<0.89 | 1 (1-1) | 1 (1-1) |
| Occupancy rate >=0.89 - <1.03 | 1.02 (0.91-1.14) | 0.98 (0.87-1.1) |
| Occupancy rate >=1.03 - <1.16 | 1.03 (0.92-1.16) | 1.00 (0.89-1.12) |
| Occupancy rate >=1.16 - <1.36 | 1.18 (1.05-1.32) | 1.09 (0.97-1.22) |
| Occupancy rate >=1.36 | 1.19 (1.06-1.34) | 1.11 (0.97-1.25) |
| **Elective surgery** |  |  |
| Reference: occupancy.rate<0.89 | 1 (1-1) | 1 (1-1) |
| Occupancy rate >=0.89 - <1.03 | 1.00 (0.86-1.16) | 1.01 (0.87-1.18) |
| Occupancy rate >=1.03 - <1.16 | 1.04 (0.90-1.21) | 1.00 (0.86-1.17) |
| Occupancy rate >=1.16 - <1.36 | 0.99 (0.86-1.16) | 0.91 (0.78-1.06) |
| Occupancy rate >=1.36 | 1.17 (1.00-1.38) | 1.10 (0.93-1.31) |
| **Low APACHE IV risk (<20%)** |  |  |
| Reference: occupancy.rate<0.89 | 1 (1-1) | 1 (1-1) |
| Occupancy rate >=0.89 - <1.03 | 1.02 (0.93-1.12) | 1.02 (0.93-1.13) |
| Occupancy rate >=1.03 - <1.16 | 1.10 (1.00-1.20) | 1.10 (1.00-1.21) |
| Occupancy rate >=1.16 - <1.36 | 1.09 (1.00-1.20) | 1.06 (0.97-1.17) |
| Occupancy rate >=1.36 | 1.17 (1.06-1.28) | 1.16 (1.05-1.29) |
| **Medium APACHE IV risk (20-70%)** |  |  |
| Reference: occupancy.rate<0.89 | 1 (1-1) | 1 (1-1) |
| Occupancy rate >=0.89 - <1.03 | 1.05 (0.98-1.11) | 1.03 (0.97-1.10) |
| Occupancy rate >=1.03 - <1.16 | 1.09 (1.02-1.17) | 1.08 (1.02-1.16) |
| Occupancy rate >=1.16 - <1.36 | 1.14 (1.07-1.22) | 1.09 (1.02-1.16) |
| Occupancy rate >=1.36 | 1.09 (1.02-1.17) | 1.08 (1.01-1.16) |
| **High APACHE IV risk (>=70%)** |  |  |
| Reference: occupancy.rate<0.89 | 1 (1-1) | 1 (1-1) |
| Occupancy rate >=0.89 - <1.03 | 1.04 (0.92-1.17) | 1.03 (0.91-1.16) |
| Occupancy rate >=1.03 - <1.16 | 1.05 (0.93-1.18) | 1.05 (0.93-1.19) |
| Occupancy rate >=1.16 - <1.36 | 1.03 (0.91-1.16) | 1.00 (0.89-1.13) |
| Occupancy rate >=1.36 | 1.00 (0.88-1.14) | 0.96 (0.84-1.09) |
| **Coronary artery bypass graft** |  |  |
| Reference: occupancy.rate<0.89 | 1 (1-1) | 1 (1-1) |
| Occupancy rate >=0.89 - <1.03 | 0.96 (0.67-1.38) | 0.95 (0.66-1.37) |
| Occupancy rate >=1.03 - <1.16 | 1.20 (0.85-1.69) | 1.11 (0.78-1.58) |
| Occupancy rate >=1.16 - <1.36 | 1.06 (0.74-1.52) | 0.87 (0.60-1.26) |
| Occupancy rate >=1.36 | 1.38 (0.96-2.00) | 1.27 (0.87-1.87) |
| **Out of hospital cardiac arrest** |  |  |
| Reference: occupancy.rate<0.89 | 1 (1-1) | 1 (1-1) |
| Occupancy rate >=0.89 - <1.03 | 1.07 (0.93-1.23) | 1.05 (0.91-1.22) |
| Occupancy rate >=1.03 - <1.16 | 1.11 (0.97-1.28) | 1.11 (0.96-1.29) |
| Occupancy rate >=1.16 - <1.36 | 1.13 (0.98-1.30) | 1.04 (0.90-1.20) |
| Occupancy rate >=1.36 | 1.02 (0.88-1.18) | 0.88 (0.75-1.04) |
| **Subarachnoid hemorrhage** |  |  |
| Reference: occupancy.rate<0.89 | 1 (1-1) | 1 (1-1) |
| Occupancy rate >=0.89 - <1.03 | 0.92 (0.60-1.41) | 1.01 (0.64-1.61) |
| Occupancy rate >=1.03 - <1.16 | 0.86 (0.55-1.35) | 0.92 (0.57-1.49) |
| Occupancy rate >=1.16 - <1.36 | 1.01 (0.64-1.60) | 0.93 (0.57-1.50) |
| Occupancy rate >=1.36 | 0.90 (0.53-1.50) | 0.85 (0.48-1.52) |
| **Oncology** |  |  |
| Reference: occupancy.rate<0.89 | 1 (1-1) | 1 (1-1) |
| Occupancy rate >=0.89 - <1.03 | 0.88 (0.70-1.09) | 0.85 (0.68-1.07) |
| Occupancy rate >=1.03 - <1.16 | 1.03 (0.83-1.28) | 0.93 (0.75-1.17) |
| Occupancy rate >=1.16 - <1.36 | 0.93 (0.74-1.16) | 0.89 (0.71-1.11) |
| Occupancy rate >=1.36 | 1.01 (0.80-1.27) | 0.93 (0.72-1.18) |
| **Trauma** |  |  |
| Reference: occupancy.rate<0.89 | 1 (1-1) | 1 (1-1) |
| Occupancy rate >=0.89 - <1.03 | 0.97 (0.78-1.19) | 0.90 (0.72-1.13) |
| Occupancy rate >=1.03 - <1.16 | 1.01 (0.82-1.26) | 1.11 (0.89-1.40) |
| Occupancy rate >=1.16 - <1.36 | 1.05 (0.84-1.31) | 1.17 (0.92-1.47) |
| Occupancy rate >=1.36 | 1.06 (0.83-1.35) | 1.11 (0.85-1.45) |
| **Intoxication** |  |  |
| Reference: occupancy.rate<0.89 | 1 (1-1) | 1 (1-1) |
| Occupancy rate >=0.89 - <1.03 | 1.00 (0.49-2.03) | 1.07 (0.45-2.55) |
| Occupancy rate >=1.03 - <1.16 | 1.41 (0.75-2.66) | 1.81 (0.83-3.93) |
| Occupancy rate >=1.16 - <1.36 | 0.78 (0.40-1.52) | 1.51 (0.70-3.27) |
| Occupancy rate >=1.36 | 0.59 (0.29-1.17) | 1.09 (0.47-2.49) |
| **Sepsis** |  |  |
| Reference: occupancy.rate<0.89 | 1 (1-1) | 1 (1-1) |
| Occupancy rate >=0.89 - <1.03 | 1.01 (0.89-1.14) | 0.95 (0.83-1.08) |
| Occupancy rate >=1.03 - <1.16 | 1.04 (0.92-1.18) | 1.02 (0.90-1.16) |
| Occupancy rate >=1.16 - <1.36 | 1.08 (0.96-1.23) | 1.01 (0.89-1.14) |
| Occupancy rate >=1.36 | 1.14 (1.01-1.30) | 1.09 (0.95-1.24) |
